# End-of-Life Cancer Care Interventions for Racially and Ethnically Diverse Populations in the USA: A Scoping Review

**DOI:** 10.3390/cancers17132209

**Published:** 2025-07-01

**Authors:** Carolyn J. Yee, Aashritha Penumudi, Terri Lewinson, Inas S. Khayal

**Affiliations:** 1 Department of Anthropology, Dartmouth College, Hanover, NH 03755, USA; carolyn.j.yee.25@dartmouth.edu; 2 Thomas Jefferson High School for Science and Technology, Alexandria, VA 22312, USA; aashri.penu@gmail.com; 3 The Dartmouth Institute for Health Policy and Clinical Practice, Geisel School of Medicine, Dartmouth College, Lebanon, NH 03756, USA; terri.d.lewinson@dartmouth.edu; 4 Biomedical Data Science, Geisel School of Medicine, Dartmouth College, Lebanon, NH 03756, USA; 5 Department of Computer Science, Dartmouth College, Hanover, NH 03755, USA; 6 Cancer Population Sciences Program, Dartmouth Cancer Center, Lebanon, NH 03756, USA

**Keywords:** end-of-life cancer care, palliative care, racial demographics, cancer disparities, health equity, racial minorities, targeted interventions

## Abstract

**Introduction**: Racial and ethnic disparities in end-of-life (EOL) cancer care persist, leading to lower rates of advance care planning (ACP), reduced access to palliative care, and poorer patient outcomes for minority populations. While previous research has documented these inequities, less is known about the specific interventions developed to address them, necessitating a comprehensive review of existing strategies aimed at improving EOL care for racial and ethnic populations. The objective of this scoping review is to examine the extent and characteristics of interventions and their outcomes designed to address racial and ethnic disparities in EOL cancer care in the United States. **Methods**: A comprehensive search of EOL cancer care interventions for minority populations was conducted in Ovid MEDLINE, CINAHL with Full Text (EBSCOhost), and Scopus (Elsevier) in September 2024. Two independent reviewers screened titles, abstracts, and full texts following the Preferred Reporting Items for Systematic Reviews and Meta-Analyses extension for Scoping Reviews (PRISMA-ScR) guidelines, with inclusion limited to studies conducted in the US and published in English. **Results**: Of 3104 screened studies, 10 met the inclusion criteria. Participants enrolled were only from Latino (n = 6 studies) or Black (n = 4 studies) populations. We identified four types of interventions, including communication skills for patients, caregivers, researchers, and clinicians (n = 2), education programs for patients (n = 1), navigation and support programs for patients and caregivers (n = 3), and training programs for health workers and community leaders (n = 4). The most effective interventions were those that addressed linguistic barriers, integrated cultural values, and involved trusted community figures. Faith-based models were particularly successful among African American patients, while bilingual navigation and family-centered ACP interventions had the greatest impact in Latino populations. **Conclusions**: This review highlights (1) the importance of culturally tailored interventions for specific minority populations and (2) the limited number of such interventions, which primarily target only the largest minority groups.

## Simple Summary

Racial and ethnic disparities in end-of-life (EOL) cancer care continue to result in lower rates of advance care planning (ACP), reduced access to palliative services, and poorer outcomes for racial and ethnic populations. While these disparities are well recognized, less is known about the specific interventions designed to address them. This review examined existing strategies aimed at improving EOL care for racial and ethnic minority patients with cancer. The most effective interventions were those that addressed language barriers, respected cultural values, and engaged trusted community members—such as lay health workers, faith leaders, and bilingual navigators. These approaches enhanced patient understanding, emotional comfort, and engagement in care planning. The findings highlight the need for culturally tailored, community-based models and offer guidance for healthcare providers, policymakers, and researchers working to ensure more equitable, compassionate, and culturally responsive EOL care for patients with advanced cancer and their families.

## 1. Introduction

End-of-life (EOL) care is a critical stage in the healthcare journey of patients and their families. It requires care that aligns with an individual’s values, beliefs, and unique needs, including considerations of language, culture, and social context [1,2,3,4]. Although EOL is a stage all living people will eventually face, it varies from person to person [5]. EOL care generally begins when a person is diagnosed with a terminal illness and has less than 6 months to live, with curative treatments no longer an option [5]. This care includes specialized services such as palliative care, advance care planning, and hospice care. Palliative care focuses on relieving pain and other symptoms, while supporting the quality of life for patients with serious illness and their families [6]. According to a Delphi panel of experts, advance care planning is defined as a process that supports adults, at any stage of health, to understand and share their personal values, life goals, and preferences for future medical care. The goal is to ensure that medical care aligns with these values and preferences during serious and chronic illness [7]. Hospice care, typically provided during the last six months of life, aims to relieve pain and suffering and support patients in achieving self-determined life closures [8].

Despite advances in medicine and an increasing emphasis on patient-centered care, significant disparities persist in EOL outcomes for racial and ethnic minority populations in the United States [9,10,11,12,13,14]. In the United States, this often includes groups like Black/African Americans, American Indians, Asians and Pacific Islanders, and Hispanics. These disparities contribute to worse healthcare outcomes for minority groups, with inequities often extending beyond the individual to affect family members and broader communities [15].

As the baby boomer generation ages, with many now 65 and older, the need to address disparities in end-of-life care becomes increasingly urgent due to the growing demand for equitable care [14,16,17]. These inequities reflect systemic barriers such as limited access to culturally sensitive care, health literacy challenges, and mistrust of the healthcare system [18,19]. To reduce these disparities, it is critical to develop and implement interventions that specifically address the unique needs of minority populations at the EOL phase. Such interventions not only improve the quality of care for individuals but also enhance the well-being of their families and communities.

Although many publications highlight the disparities in EOL care, less is known about the specific interventions developed to address these inequities for patients with cancer and their families. Existing studies often focus on individual patient-level factors, such as literacy and cultural norms, or explore structural elements like language access and community engagement [13]. However, there is a need for a comprehensive synthesis of the types, characteristics, and outcomes of these interventions to better understand the most effective strategies to support EOL discussions and care delivery for patients with cancer from minority populations.

This scoping review aims to address this gap by systematically mapping the literature on interventions designed to reduce disparities in EOL cancer care for racial and ethnic minority populations in the United States. Using Peter and Godfrey’s framework for systematic scoping reviews, along with validated methodological additions, this review provides a broad examination of existing evidence [20]. This scoping review seeks to summarize and disseminate research findings, examine the extent and range of existing interventions, and identify critical gaps in the literature. Ultimately, this work aims to guide future efforts in creating and implementing equitable, culturally sensitive interventions for minority populations at the EOL stage of cancer care.

## 2. Methods

This scoping review was conducted following the methodology described in Levac [21] and the Preferred Reporting Items for Systematic reviews and Meta-Analyses extension for Scoping Reviews (PRISMA-ScR) Checklist [22]. See Appendix A for the PRISMA-ScR checklist.

### 2.1. Search Strategy

We conducted searches in Ovid MEDLINE (PubMed), CINAHL with Full Text (EBSCOhost), and Scopus (Elsevier) from the inception of each database to 3 September 2024, using controlled vocabulary and keywords with synonyms that reflected the concepts of interventions, cancer, health equity, and palliative care. The search strategy was developed collaboratively by CJY and ISK and incorporated an initial set of keywords and index terms, which were refined following a comprehensive search strategy targeting cancer-related disparities and interventions [23]. See Appendix B for the final search keywords.

### 2.2. Screening for Study Eligibility

Studies were included in our review if they met the following five criteria. First, studies exclusively included U.S.-based populations. Second, studies included an exclusive focus on patients with cancer. Papers focused on caregivers, healthcare providers, or community health workers for patients with cancer were included only if the paper met all remaining criteria. Third, studies included a focus on EOL cancer care. Fourth, interventions must address disparities in care for populations of racial and ethnic demographics. Fifth, they had to explicitly describe an intervention related to EOL care services aimed at addressing a healthcare challenge, improving outcomes, or modifying patient care processes. If the description of an intervention was ambiguous or lacked sufficient detail to determine its implementation or intended purpose, the paper was excluded.

Search results were downloaded to Rayyan (www.rayyan.ai), and duplicate records were first removed via Rayyan’s automatic duplicate detection and then manually. Titles and abstracts were independently screened for eligibility by two reviewers (CJY and AP). Screening discrepancies were jointly resolved or by involving an additional reviewer (ISK).

### 2.3. Data Extraction

A draft extraction form was piloted based on the Data Collection Form in the Cochrane Handbook Table 21.5.a PerSPecTIF [24] and revised as necessary during the process. We extracted study characteristics, patient population information, study context details, intervention implementation details, outcomes, and challenges and facilitators. Data extraction was performed independently by two reviewers (CJY and AP), with discrepancies resolved by discussion or consultation with a third reviewer (ISK).

### 2.4. Data Analysis

We summarized the results descriptively, presenting data in compiled figures when appropriate. We synthesized the extracted data across studies by Intervention Design, Intervention Tailoring for Racial and Ethnic Populations, Intervention Implementation Challenges, and Intervention Outcomes. We categorized Intervention Designs in three steps. First, we extracted the text representing the intervention design for all studies. Second, we identified to whom the intervention was applied (e.g., patients, caregivers, clinicians, and/or researchers). Third, we identified and categorized the terminology used in the text (e.g., communication coaching, conversation guide, etc.). The Intervention Design categories, their description, and exemplar quotes from each paper are described in Appendix C. A similar process was applied to categorizing Intervention Tailoring for racial and ethnic populations, as described in Appendix D. For Intervention Implementation Challenges and Intervention Outcomes, we identified and reported the specifically described challenges and outcomes for each study.

## 3. Results

**Figure 1:**
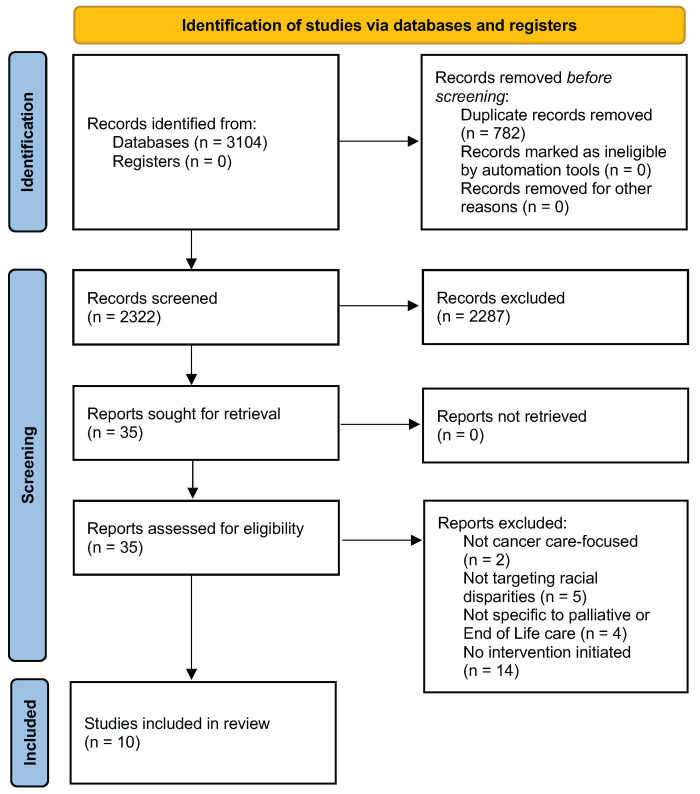
Preferred Reporting Items for Systematic Reviews and Meta-Analyses registers (PRISMA) flowchart depicting studies from the following databases: Ovid MEDLINE (PubMed), CINAHL with Full Text (EB-SCOhost), and Scopus (Elsevier).

Our search yielded 3104 records identified from databases. Figure 1 showcases our result records in a PRISMA flow diagram. We removed 782 duplicate records. We then screened the titles and abstracts of the remaining 2322 records. We identified a total of 35 papers for eligibility. Following a full-text review, we included a total of 10 papers in this scoping review.

### 3.1. Study Characteristics

For each of the 10 studies, Table 1 provides a summary of study information, patient population information, cancer information, and intervention implementation factors. Approximately 942 participants were included across these 10 studies. Studies were predominantly focused on single ethnic–racial populations, with the majority of studies identifying participants as African American or Black (n = 4 studies) [25,26,27,28], or Latino (n = 6 studies) [29,30,31,32,33,34], published between 2013 and 2023. Studies were conducted in Black and African American communities in North Carolina, South Carolina, Maryland, and Alabama. Studies were conducted with LatinX populations from Colorado, North Carolina, and Massachusetts. Studies evaluated interventions in various healthcare settings, including hospitals, hospices, and community-based care facilities. Two studies were randomized control trials [27,30], and the remaining eight studies were pilot studies; specifically a feasibility study (n = 1) [29], evaluation studies (n = 3) [25,28,32], pre-post studies (n = 2) [26,31], and intervention development studies (n = 2) [33,34].

### 3.2. Intervention Design

We identified 4 categories of interventions. These included *Communication Skills Programs for Patients, Caregivers, Researchers, and Clinicians* (n = 2) [28,34], *Education Programs for Patients* (n = 1) [33], *Navigation and Support Programs for Patients and Caregivers* (n = 3) [26,29,30], and *Training Programs for Health Workers and Community Leaders* (n = 4) [25,27,31,32]; see Table 2.

First, *communication skills programs for patients, caregivers, researchers, and clinicians* were defined as interventions that provided structured, culturally competent communication coaching and tools to support advance care planning (ACP) across patients, caregivers, researchers, and clinicians. These interventions integrated sociocultural frameworks and patient-centered language to promote values-based discussions about prognosis, goals, and care preferences. The programs also emphasized practical skills like role-play, scripting, and relational framing to improve participants’ confidence and ability to engage in sensitive conversations.

Second, *education programs for patients* were defined as interventions that utilized bilingual materials, culturally relevant messaging, and plain-language resources to improve patient and caregiver understanding of ACP, symptom management, and palliative care options. These programs adapted educational content to align with specific cultural beliefs, family dynamics, and decision-making processes, addressing literacy barriers and ensuring accessibility for diverse populations.

Third, *navigation and support programs for patients and caregivers* were defined as interventions that provided logistical assistance, emotional support, and healthcare system guidance to both patients and their caregivers navigating complex EOL care decisions. These programs typically included bilingual patient navigators, culturally tailored decision aids, and assistance with ACP documentation, ensuring that minority patients received equitable access to palliative care services.

Fourth, *training programs for health workers and community leaders* were defined as capacity-building interventions that provided education and skill development to community health workers, lay health advisors, and faith leaders to equip them with the tools necessary to support EOL care discussions, advocate for palliative care engagement, and facilitate ACP planning. These interventions emphasized the role of trusted community figures in bridging healthcare access gaps and enhancing culturally competent care delivery.

### 3.3. Intervention Tailoring for Racial and Ethnic Populations

All end-of-life cancer care interventions were tailored to the unique racial and ethnic study population. Specifically, we identified intervention tailoring for *language, literacy, cultural values, faith-based values, and trust*.

For Latino populations (n = 6), interventions incorporated a range of culturally tailored strategies to address language, literacy, and cultural values. The Planning for your Advanced care Needs (PLAN) intervention, developed by Shen et al. [34], provided a structured manual grounded in communication competence and sociocultural theory to support Latino patients with advanced cancer in discussing ACP, highlighting the need for explicit communication scripts to guide Latino patients in end-of-life care discussions. Similarly, Bekelman et al. [29] described the Apoyo con Cariño program, a patient navigator intervention in which bilingual lay health workers engaged Latino patients with advanced cancer in home-based discussions on palliative care and ACP, emphasizing culturally tailored communication and decision-making support. Bekelman et al. [29] found that integrating cultural values such as familismo improved engagement in ACP in connecting with the participants. As one participant reported in discussing the video vignettes shown as part of the program, “I can’t remember a video that I couldn’t relate to. I cried so much watching them because it’s a reality and we know cancer is affecting us that way.” (pg. 693)

For African American populations (n = 4), trust and faith-based values in interventions played a key role in addressing spiritual needs and in reinforcing trust in the healthcare system. Hanson et al. [26] and Sanders et al. [28] reported that culturally congruent interventions that incorporated spiritual and religious elements fostered greater trust in palliative care. One participant stated, “I want others to know my need so they can better understand my needs. Circles of Care members are now more like family members.” (pg. 296) [26]. These programs emphasized peer support and community engagement as central components in improving advance directive completion rates. In one study by Sanders et al. [28], intervention tailoring included literacy as well as faith-based values.

### 3.4. Intervention Implementation Challenges

For each of the 10 studies, Table 2 identifies common barriers and facilitators for each intervention, categorized by patient racial demographics. Common barriers influencing implementation included resource constraints as a result of recruitment challenges due to concerns of privacy and inconsistent responses [25,26,28,29,30,31,32,33,34], and perceived cultural and trust limitations across racial demographics of participants and clinicians/caregivers [25,26,28]. Common facilitators influencing implementation included involvement of trusted community leaders, such as pastors or community health workers, facilitating trust-building and program acceptance [25,28]. Family-centered approaches aligned with cultural values for both Latino and African American populations [26,29].

This review identified systemic barriers that hinder the implementation and scalability of interventions. Recruitment challenges were a consistent theme, differing across racial demographics [26,27,28]. Among Latino populations, systemic unfamiliarity creates hesitancy in engaging with the healthcare system. Additionally, cultural values such as familismo, which emphasize keeping decisions within the family, often lead to delayed discussions around ACP [29,34]. Among African American populations, they more often face barriers tied to a historical mistrust of the healthcare system, stemming from systemic racism and historically unethical medical practices [25,28]. Tailoring interventions to account for these culturally and historically grounded barriers is essential to improving equitable EOL care delivery.

Additionally, resource constraints, including the availability of culturally competent staff and funding for long-term sustainability, were frequently cited [25,28,33]. Faith-based programs and community-led initiatives were effective in addressing mistrust, but often relied on volunteer networks, limiting their scalability [25,28].

### 3.5. Intervention Outcomes

Interventions demonstrated varying levels of success in *improving palliative care knowledge and communication*, *improving ACP engagement*, and *improving patient satisfaction and trust in care*.

*Improved palliative care knowledge and communication*: Nine studies demonstrated improvements in palliative care knowledge and communication confidence among patients, caregivers, or community health workers following culturally tailored interventions [25,26,27,28,29,31,32,33,34]. In Johnson et al., participants reported greater ease initiating conversations with family and providers [31]. Gains in knowledge were not abstract or isolated, but were immediately applied to interpersonal and community-based decision-making. Similarly, in Leiter et al., Latino patients reported that the culturally adapted video-based intervention helped them express their values and improved their understanding of chemotherapy and ACP. Patients described the videos as relatable and helpful in understanding treatment and ACP concepts, particularly when reflecting their cultural values and personal experiences [33]. This sense of increased understanding was echoed in Monton et al. [27], where African American community health workers completed a palliative care training program and demonstrated improved knowledge in palliative care concepts, symptom management, and ACP support. Participants described the training as filling knowledge gaps and improving their preparedness for medical decision-making [27]. These findings suggest that tailored, community-engaged interventions can enhance palliative care literacy and increase communication efficacy among both patients and those delivering care in underserved populations.

*Improved ACP engagement*: Among Latino populations, Fischer et al. [30] and Shen et al. [34] reported improvements in ACP engagement through culturally tailored interventions. Fischer et al. [30] showed that lay navigators supported increased ACP documentation among Spanish-speaking patients, and Shen et al. [34] described how the PLAN manual helped Latino patients feel more prepared to initiate conversations about advance care preferences. Among African American populations, Sanders et al. [28] and Hanson et al. (2013) [25] emphasized faith-based and community-engaged interventions as effective for fostering ACP discussions by addressing mistrust in the healthcare system. Hanson et al. (2014) [26] reported improvements in ACP documentation using a culturally adapted video aid, though it did not involve a faith-based model.

ACP engagement emerged as a central improved outcome across four studies [29,30,32,34] particularly where interventions used culturally and linguistically tailored approaches. Rather than functioning as isolated tools, these interventions leveraged trusted relationships, culturally resonant communication, and practical support systems to move beyond awareness and toward concrete ACP behaviors. In Bekelman et al., bilingual lay health worker support yielded a statistically significant increase in ACP documentation among Latino patients, highlighting the power of sustained, language-congruent support to transform patient readiness into action [29]. Fischer et al. [30] also found that lay navigators increased ACP documentation among Spanish-speaking patients, and Shen et al. [34] described increases in ACP behaviors following the PLAN manual intervention. These studies demonstrated that patients guided by trained lay health workers were more likely to articulate care goals and engage in ACP discussions, reinforcing the value of culturally familiar intermediaries.

In African American communities, ACP engagement was also linked to cultural congruence and spiritual trust. Hanson et al. (2013) found that participants in a church-based intervention felt more comfortable talking about the end of life in a church setting, underscoring the effectiveness of trusted community institutions in bridging gaps in care planning [25]. Together, multiple studies emphasized that ACP engagement was enhanced through community-based, linguistically adapted, and culturally tailored strategies, particularly when delivered in trusted settings. Interventions that considered cultural values, community trust, and communication preferences were more likely to support patient readiness and willingness to engage in end-of-life conversations. Studies incorporating structured ACP discussions, bilingual navigation services, and multimedia educational tools were associated with increased patient understanding of ACP and increased completion of advance directives, as well as greater patient-reported readiness for end-of-life planning.

Across racial demographics, Johnson et al. [31] and Sanders et al. [28] demonstrated that Lay Health Workers (LHWs), when trained in culturally adapted communication strategies, helped address both informational and emotional barriers to ACP by facilitating trust-based conversations in community settings. Both Johnson et al. [31] and Sanders et al. [28] noted that lay facilitators were effective in initiating ACP conversations within historically underserved communities, emphasizing the promise of community-based approaches for increasing culturally aligned care access.

*Improved patient satisfaction and trust in care*: Improved patient satisfaction and trust in care emerged as a consistent outcome across five studies [25,29,30,31,34], with interventions that prioritized cultural congruence, emotional support, and trusted communication channels. In Fischer et al., [30] Latino patients participating in the Apoyo con Cariño navigation program described feeling emotionally supported and more prepared for decision-making, highlighting how patient navigation tailored to cultural values can alleviate uncertainty and distress. A subsequent study by Bekelman et al., using the same cohort, reinforced this conclusion: patients rated the program as helpful and aligned with their values, suggesting that bilingual navigation not only improved outcomes but also deepened satisfaction with care delivery through relational trust and cultural alignment [29,30].

This pattern extended across other settings and populations. In church-based interventions, African American participants described the faith-centered model as comforting and respectful of their spiritual needs [25], reinforcing the centrality of community-rooted approaches in cultivating trust. The familiarity of the church setting served as a powerful facilitator for comfort and confidence in EOL care discussions.

Collectively, these studies suggest that trust and satisfaction are achieved most effectively through interventions that recognize and integrate the cultural, familial, and spiritual dimensions of patients’ lives.

Overall, multiple studies have reported that interventions addressing linguistic barriers, integrating cultural values, and involving trusted community figures—such as bilingual navigation and community-based facilitators—improved patient engagement and comfort with ACP and EOL care. Faith-based models were shown to be particularly effective among African American patients, while in Latino populations, bilingual navigation and family-centered ACP interventions had the greatest impact in Latino populations. The findings highlight the importance of culturally competent EOL care strategies associated with improved communication, ACP engagement, and emotional comfort among racial and ethnic minority patients.

## 4. Discussion

Research findings from this scoping review reveal the wide variety of interventions developed to address disparities in end-of-life (EOL) cancer care for racial and ethnic minority populations in the United States. These interventions include communication skills programs for patients, caregivers, researchers, and clinicians [28,34]; education programs for patients [33]; navigation and support programs for patients and caregivers [26,29,30]; and training programs for health workers and community leaders [25,27,31,32]. Across these programs, our findings reveal two critical points: (1) the importance of tailoring interventions to specific racial and ethnic population needs, and (2) the very limited number of end-of-life interventions for patients with cancer—only for the two largest minority populations.

A key finding from this review is the importance of culturally tailored end-of-life interventions for patients with cancer among minority populations. Many studies emphasized that Latino and African American patients often face significant cultural and linguistic barriers to ACP, leading to lower rates of advance directive completion and higher rates of unwanted aggressive end-of-life care. The need for cultural tailoring in palliative care is thus underscored in these studies, aligning with the existing literature on palliative care in minority communities describing the importance of culturally tailored palliative care [35,36,37,38]. In the studies found in this scoping review, programs such as “Puente para cuidar” and other patient navigation services have demonstrated the importance of aligning care delivery with cultural values, including familismo in Latino communities and spiritual guidance in African American populations. Tailored educational tools incorporating plain language and bilingual content were effective in improving health literacy and care engagement, particularly among patients with limited English proficiency.

From these results, it becomes clear that ACP engagement is fundamentally a social practice, not a purely informational or procedural one. The studies in this review highlight that what prompts people to engage in conversations about death is rarely just content—it is context [39]. The most successful interventions did not simply teach patients what to do, but instead transformed the conditions under which patients felt safe enough to act. In this way, ACP becomes not just an outcome of culturally tailored interventions but a proxy for relational trust, psychological safety, and institutional legitimacy. These findings challenge the notion that ACP can be achieved solely through standardized educational materials or documentation prompts. Instead, they underscore that ACP is a relational and cultural achievement, requiring not just knowledge, but resonance, delivered by those who understand, belong, and are trusted. To scale ACP effectively in diverse populations, healthcare systems must center community leadership, cultural fluency, and emotional accessibility as essential components of serious illness care. This relational framing of ACP aligns with a growing body of scholarship that redefines ACP as a fundamentally social practice. Sudore and Fried argue that the goal of ACP should shift from the completion of advance directives to the preparation of patients and their families for complex, real-time decision-making [40]. They emphasize that effective ACP requires iterative, emotionally grounded conversations that reflect patients’ evolving values and relationships. Similarly, Rietjens et al., in an international consensus statement, define ACP as an ongoing process embedded within interpersonal relationships, recommending that it include not only the individual patient, but also family members and healthcare professionals in sustained dialogue [41].

Moreover, scholars of relational autonomy have long critiqued individualistic models of ACP for neglecting the influence of social context, power dynamics, and communal identity on end-of-life decision-making [42,43]. These relational perspectives support our finding that ACP operates less as a discrete medical task and more as a social and ethical process, shaped by cultural meanings, institutional legitimacy, and interpersonal trust. Together, this literature reinforces the conclusion that if ACP is to be meaningfully and equitably scaled, it must be embedded in social relationships and community networks, rather than treated as a standalone educational or legal intervention.

The emphasis on community-based and lay-delivered models (e.g., lay health workers in Leiter et al. [33] and church leaders in Hanson et al. 2013 [25]) reflects a broader critique of the traditional medical model: that institutional authority alone is insufficient to catalyze behavior change, particularly in populations with long histories of marginalization or medical neglect. ACP conversations are more likely to occur when they are relationally mediated: when the messenger looks like the patient, speaks the same language, and understands the patient’s cultural scripts around family, illness, and mortality. The Institute of Medicine’s Dying in America report underscores that communication about serious illness is only effective when it is emotionally resonant and culturally competent, calling for care that aligns with the preferences of both patients and families [44].

Moreover, the relational nature of ACP in these interventions suggests that health systems must reimagine who holds power in care conversations. The most effective messengers were not physicians, but peer educators, pastors, lay health workers, and bilingual navigators: individuals with emotional proximity to patients and the credibility of lived experience. This highlights a broader structural implication: if we want equitable ACP outcomes, we must invest not just in clinical tools but in community infrastructure. This finding is supported by the literature on shared decision-making and power dynamics in healthcare. Shared decision-making has long been positioned as a corrective to traditional medical hierarchies, which often center authority with the clinician and marginalize the patient’s voice. In contrast, shared decision-making reframes patients not as passive recipients of care, but as active participants in the clinical decision-making process. Scherer et al. demonstrate that perceived power asymmetry and embarrassment significantly impede patient participation and elevate decisional conflict, especially among structurally marginalized groups, underscoring the need to dismantle hierarchical barriers for effective engagement [45]. Meanwhile, community health worker-led decision coaching has emerged as a powerful mechanism to redistribute authority and empower patients, particularly in populations with a history of distrust in medical systems. For instance, community health worker interventions in prostate cancer screening have significantly improved shared decision-making in Black male patients by bridging cultural and relational divides [46].

However, the effectiveness of cultural tailoring also highlights a gap in standardization across interventions. For example, while some studies emphasized family-centered care and bilingual navigation services, others focused more narrowly on logistical barriers to care. Without a clear taxonomy for defining cultural tailoring or measuring its impact, it is challenging to compare outcomes across interventions or identify best practices for scalability. Recent work in health promotion offers some direction: Kreuter and colleagues’ typology differentiates between surface-level tailoring (e.g., language translation, culturally familiar imagery) and deep-structure adaptations (e.g., aligning interventions with core cultural values or social norms) [47]. Furthermore, Baker et al. suggest that while tailored interventions can improve outcomes, their effects can vary substantially depending on how tailoring is designed and delivered [48]. This highlights a key finding: that in order to scale culturally grounded ACP, we need not only to invest in culturally concordant delivery but also to develop standardized frameworks that define and measure cultural adaptation, enabling us to identify which elements matter most and replicate them reliably at scale.

This review identified a very limited number of end-of-life interventions for patients with cancer—only for the two largest minority populations, Latino and Black/African American. No studies were identified for specific populations, including Asians, American Indians, Alaska Natives, Native Hawaiians, Other Pacific Islanders, Arab Americans, or mixed populations, or for multiple populations. Other scoping and systematic reviews examining interventions for racial and ethnic minorities also found comparable numbers of included papers [23,49,50]. These reviews highlight both the persistent scarcity of rigorously evaluated interventions in this space and the urgent need for more targeted research and funding to support culturally responsive models of end-of-life care. While many studies focused on improving ACP and symptom management, hospice utilization remains disproportionately low among racial and ethnic minority populations. Expanding the role of culturally tailored patient navigation, community health worker-led support programs, and faith-based engagement within hospice care may improve access to and trust in EOL services. Additionally, policy changes that support Medicaid and Medicare reimbursement for culturally tailored palliative care programs could enhance the sustainability of these interventions. Greater investment in multilingual, low-literacy educational tools and workforce training for culturally competent palliative care professionals could also improve EOL care experiences for minority populations.

This scoping review has limitations. The studies included were limited to those conducted in the United States, excluding potentially relevant international research. Given that many countries have well-developed palliative care systems, examining global interventions could provide valuable insights into addressing disparities in the U.S. context. Future research may consider cross-national comparisons to identify best practices in culturally tailored end-of-life care. Second, most interventions were not designed for scalability, and many studies lacked longitudinal data to assess long-term outcomes. Ensuring that successful interventions can be implemented across different healthcare settings and geographies remains a challenge.

## 5. Conclusions

This scoping review highlights the importance of culturally tailored interventions in addressing disparities in end-of-life cancer care. Effective strategies were those that were culturally tailored through patient navigation programs, community-driven models, and tailored educational tools, all of which improve care engagement and patient satisfaction. However, systemic barriers, including resource constraints and a lack of standardization, hinder broader implementation. Future research should prioritize scalable, evidence-based interventions. Expanding culturally tailored approaches within hospice and palliative care settings, integrating community health workers, and enhancing language accessibility can help bridge existing gaps. Establishing clear definitions, standardized metrics, and longitudinal assessments will be essential in advancing this field and ensuring equitable EOL care for all populations.

## Figures and Tables

**Table 1:** Study results by study information, patient population information, cancer information, and intervention implementation factors (GU = genitourinary; GI = gastrointestinal; GYn = gynecologic; HS = high-school level).

Authors	Age	Population Location	Minority Group	Setting of the Study	Education	Income	Specific Type	Advanced Cancer?	Language	Intervention Types	What Was Tailored for?	Challenges	Outcomes
Hanson et al. 2013	2%: <21, 2%: 21–30, 4%: 31–40, 19%: 41–50, 21%: 51–60, 17%: 61–70, 28%: >71, 6%: not disclosed	Central North Carolina	98%: Black, 2%: Hispanic	Community settings	Not specified	Not specified	Not specified	Exclusively Advanced Cancer Patients	English Only	Training Programs for Health Workers and Community Leaders	Trust and Faith-based Values	Cultural and Trust Issues, Resource Constraints	Improved Palliative Care Knowledge and Communication, Improved Patient Satisfaction and Trust in Care
Hanson et al. 2014	13%: <41, 13%: 41–50, 38%: 51–60, 38%: >61	North Carolina	African American	Academic medical center and affiliated hospice program	Not specified	Not specified	Not specified	Mostly (64%) Advanced Cancer Patients	English Only	Navigation and Support Programs for Patients and Caregivers	Trust and Faith-based Values	Cultural and Trust Issues, Resource Constraints	Improved Palliative Care Knowledge and Communication
Monton et al. 2023	Not specified	Maryland and Alabama	African American	Johns Hopkins Hospital, TidalHealth Peninsula Regional and University of Alabama at Birmingham Hospital	Not specified	Not specified	Not specified	Exclusively Advanced Cancer Patients	English Only	Training Programs for Health Workers and Community Leaders	Trust and Faith-based Values	Recruitment and Retention	Improved Palliative Care Knowledge and Communication
Sanders et al. 2023	Mean age = 71 years (range 50–88)	Charleston, South Carolina	African American	Church-based and community volunteer settings, including home visits	39%: <HS, 26%: HS, 17%: some college, 17%: college graduate	65%: <10k, 13%: 10–20k, 4%: 20–40k, 4%: 60–70k, 4%: >75k	Lung (9%), GU (13%), GYN (30%), GI (22%), Glioblastoma (4%), Other (22%)	Exclusively Advanced Cancer Patients	English Only	Communication Skills Programs for Patients, Caregivers, Researchers, and Clinicians	Literacy and Faith-based Values	Cultural and Trust Issues, Resource Constraints	Improved Palliative Care Knowledge and Communication
Bekelman et al. 2020	Mean: 58 years (SD ± 13)	Rural Eastern Colorado	Latinx	Safety-net clinics, community cancer clinics, National Cancer Institute, and home visits	36%: <HS	50%: <15k, 50%: >15k	Breast (16%), GI (21%), GU (21%), Lung (21%), Other (21%)	Exclusively Advanced Cancer Patients	Bilingual (English/Spanish)	Navigation and Support Programs for Patients and Caregivers	Language, Literacy, and Cultural Values	Resource Constraints	Improved Palliative Care Knowledge and Communication, Improved ACP Engagement, Improved Patient Satisfaction and Trust in Care
Fischer et al. 2018	Mean: 58.1 years (SD ± 13.6)	Rural Eastern Colorado	Latinx	Safety-net clinics, community cancer clinics, National Cancer Institute, and home visits	50.2%: <HS	53.6%:<15k, 88.2%: Unemployed	Breast (19.3%), GI (33.6%), GU (11.7%), GYN (10.3%), Lung (9.0%), Hematologic (7.6%), Other (8.5%)	Exclusively Advanced Cancer Patients	Bilingual (English/Spanish)	Navigation and Support Programs for Patients and Caregivers	Language, Literacy, and Cultural Values	Resource Constraints	Improved ACP Engagement, Improved Patient Satisfaction and Trust in Care
Johnson et al. 2022	Mean: 41.2 years (SD ± 13.3)	Rural North Carolina	Latinx	Over the phone, location not specified	40%: HS	73%: Worked full time	Not specified	Exclusively Advanced Cancer Patients	Bilingual (English/Spanish)	Training Programs for Health Workers and Community Leaders	Language, Literacy, and Cultural Values	Resource Constraints	Improved Palliative Care Knowledge and Communication, Improved Patient Satisfaction and Trust in Care
Larson et al. 2023	Patients: Mean age = 51 years (SD ± 14)	North Carolina	Latinx	Regional cancer centers in Eastern North Carolina	71.4%: <HS, 14.3%: HS, 14.3%: >HS	85.7%: Unemployed, 14.3%: Employed, specified as low income	Breast (28.6%), Hematological (25.7%), GI (20%), GU/GYN (14.3%), Lung (5.7%), Head and Neck (5.7%)	Exclusively Advanced Cancer Patients	Bilingual (English/Spanish)	Training Programs for Health Workers and Community Leaders	Language, Literacy, and Cultural Values	Resource Constraints	Improved Palliative Care Knowledge and Communication, Improved ACP Engagement
Leiter et al. 2023	Older Adult patients	Massachusetts	Latinx	Clinical settings - Dana Farber Cancer Institute and Boston Medical Center	Not specified	Not specified	All GI	Exclusively Advanced Cancer Patients	Bilingual (English/Spanish)	Education Programs for Patients that are Culturally Tailored	Language, Literacy, and Cultural Values	Resource Constraints	Improved Palliative Care Knowledge and Communication
Shen et al. 2023	Mean Patient age = 53.4 years (SD ± 17.5)	Northeast academic medical center in an urban setting	Latinx	Intervention completed at home	54.5%: <HS, 18.2%: Some College, 27.3%: College or more	45.4%: <40k, 27.3%: >40k, 27.3%: did not disclose	Not specified	Exclusively Advanced Cancer Patients	Bilingual (English/Spanish)	Communication Skills Programs for Patients, Caregivers, Researchers, and Clinicians	Language, Literacy, and Cultural Values	Language and Health Literacy Barriers, Resource Constraints	Improved Palliative Care Knowledge and Communication, Improved ACP Engagement, Improved Patient Satisfaction and Trust in Care

**Table 2:** Study results presented by patient racial group, for Intervention Design, Intervention Tailoring, Intervention Implementation Challenges, and Intervention Outcomes.

Racial Group & Studies (n)	Intervention Categories	Intervention Tailoring	Implementation Challenges	Intervention Outcomes
**African American/ Black Populations** Hanson 2013, Hanson 2014, Monton, Sanders **(4)**	Communication Skills Programs for Patients, Caregivers, Researchers, and Clinicians (**Sanders**) Education Programs for Patients that are Culturally Tailored (None) Navigation and Support Programs for Patients and Caregivers (**Hanson 2014**) Training Programs for Health Workers and Community Leaders (**Hanson 2013, Monton**)	Trust and Faith-based Values (**Hanson 2013, Hanson 2014, Monton**) Literacy and Faith-based Values (**Sanders**) Language, Literacy, and Cultural Values (None)	Cultural and Trust Issues (**Hanson 2013, Hanson 2014, Sanders**) Resource Constraints (**Hanson 2013, Hanson 2014, Sanders**) Recruitment and Retention (**Monton**) Language and Literacy Barriers (None)	Improved Palliative Care Knowledge and Communication (**Hanson 2013, Hanson 2014, Monton, Sanders**) Improved ACP Engagement (None) Improved Patient Satisfaction and Trust in Care (**Hanson 2013**)
**Latino/Latinx Populations** Bekelman, Fischer, Johnson, Larson, Leiter, Shen **(6)**	Communication Skills Programs for Patients, Caregivers, Researchers, and Clinicians (**Shen**) Education Programs for Patients that are Culturally Tailored (**Leiter**) Navigation and Support Programs for Patients and Caregivers (**Bekelman, Fischer**) Training Programs for Health Workers and Community Leaders (**Johnson, Larson**)	Trust and Faith-based Values (None) Literacy and Faith-based Values (None) Language, Literacy, and Cultural Values (**Bekelman, Fischer, Johnson, Larson, Leiter, Shen**)	Cultural and Trust Issues (None) Resource Constraints (**Bekelman, Fischer, Johnson, Larson, Leiter, Shen**) Recruitment and Retention (None) Language and Literacy Barriers (**Shen**)	Improved Palliative Care Knowledge and Communication (**Bekelman, Johnson, Larson, Leiter, Shen**) Improved ACP Engagement (**Bekelman, Fischer, Larson, Shen**) Improved Patient Satisfaction and Trust in Care (**Bekelman, Fischer, Johnson, Shen**)

## Data Availability

All data are available in the manuscript and appendices.

## Appendix A. Preferred Reporting Items for Systematic Reviews and Meta-Analyses Extension for Scoping Reviews (PRISMA-ScR) Checklist [22]

**Section**	**Item**	**PRISMA-ScR Checklist Item**	**Reported on Page #**
Title	1	Identify the report as a scoping review.	1
Abstract	2	Provide a structured summary that includes (as applicable): background, objectives, eligibility criteria, sources of evidence, charting methods, results, and conclusions that relate to the review questions and objectives.	2
Introduction	3	Describe the rationale for the review in the context of what is already known. Explain why the review questions/objectives lend themselves to a scoping review approach.	3
Objectives	4	Provide an explicit statement of the questions and objectives being addressed with reference to their key elements (e.g., population or participants, concepts, and context) or other relevant key elements used to conceptualize the review questions and/or objectives.	3
Methods	5	Indicate whether a review protocol exists; state if and where it can be accessed (e.g., a Web address); and if available, provide registration information, including the registration number.	4
Eligibility Criteria	6	Specify characteristics of the sources of evidence used as eligibility criteria (e.g., years considered, language, and publication status), and provide a rationale.	4
Information Sources	7	Describe all information sources in the search (e.g., databases with dates of coverage and contact with authors to identify additional sources), as well as the date the most recent search was executed.	4
Search	8	Present the full electronic search strategy for at least 1 database, including any limits used, such that it could be repeated.	19
Selection of Sources of Evidence	9	State the process for selecting sources of evidence (i.e., screening and eligibility) included in the scoping review.	4
Data Charting Process	10	Describe the methods of charting data from the included sources of evidence (e.g., calibrated forms or forms that have been tested by the team before their use, and whether data charting was done independently or in duplicate) and any processes for obtaining and confirming data from investigators.	5
Data Items	11	List and define all variables for which data were sought and any assumptions and simplifications made.	4–5
Critical Appraisal of Individual Sources of Evidence	12	If done, provide a rationale for conducting a critical appraisal of included sources of evidence; describe the methods used and how this information was used in any data synthesis (if appropriate).	n/a
Synthesis of Results	13	Describe the methods of handling and summarizing the data that were charted.	4–5
Results	14	Give numbers of sources of evidence screened, assessed for eligibility, and included in the review, with reasons for exclusions at each stage, ideally using a flow diagram.	4–5
Characteristics of Sources of Evidence	15	For each source of evidence, present characteristics for which data were charted and provide the citations.	5–11
Critical Appraisal Within Sources of Evidence	16	If done, present data on critical appraisal of included sources of evidence (see item 12).	n/a
Results of Individual Sources of Evidence	17	For each included source of evidence, present the relevant data that were charted that relate to the review questions and objectives.	5–11
Synthesis of Results	18	Summarize and/or present the charting results as they relate to the review questions and objectives.	5–11
Discussion	19	Summarize the main results (including an overview of concepts, themes, and types of evidence available), link to the review questions and objectives, and consider the relevance to key groups.	11
Limitations	20	Discuss the limitations of the scoping review process.	15
Conclusions	21	Provide a general interpretation of the results with respect to the review questions and objectives, as well as potential implications and/or next steps.	15
Funding	22	Describe sources of funding for the included sources of evidence, as well as sources of funding for the scoping review. Describe the role of the funders of the scoping review.	16

## Appendix B. Search Strategy Keywords Applied to Ovid MEDLINE (PubMed), CINAHL with Full Text (EBSCOhost), and Scopus (Elsevier)

Ovid MEDLINE
1. Implementation Science/ or (implement* or intervent* or qual* or program* or intermediat* or administ* or navigat*).ti,ab,kf AND 2. Neoplasms/ or cancer* or carcinoma* or carcinoid* or cyst* or lesion* or malignan* or metastas* or metastat* or neoplas* or oncol* or tumor* or tumour*).ti,ab,kf Or (adenocarcinoma* or astrocytoma* or blastoma* or carcinosarcoma* or chondrosarcoma* or choriocarcinoma* or craniopharyngioma* or ependymoma* or erythroleukem* or erythroleukaem* or fibrosarcoma* or glioblastoma* or glioma* or hepatocarcinoma* or histiocytoma* or hodgkin* or leukemi* or leukaemi* or lymphoma* or lymphosarcoma* or macroglobulinemi* or melanoma* or medulloblastoma* or meningioma* or mesothelioma* or myeloma* or myelodysplastic or myeloproliferative or nephroblastoma* or neuroblastoma* or osteosarcoma* or pheochromocytoma* or pineoblastoma* or retinoblastoma* or rhabdomyosarcoma* or sarcoma* or thymoma*).ti,ab,kf AND 3. Health Equity/ or Healthcare Disparities/ or Minority Health/ or exp health inequities/ or (prejudice* or inequal* or inequit* or disparit* or equalit* or equit* or unequal* or injustice* or justice* or racial or racist* or ethnicit* or racism* or stigma* or sterotyp*).ti,ab,kf Or ((implicit or health or conscious or unconscious or nonconscious or non-conscious) adj3 (attitude* or bias*)).ti,ab,kf Or ((minorit* or structural* or cultural* or multi-cultur* or multicultur* or linguistic* or social* or intercultural* or inter-cultural* or cross-cultural or crosscultural or multi-rac* or multi-ethnic* or multiethnic* or multi-lingual or multilingual or trans-cultural or transcultural or ethnocultur* or ethnocultur* or socio-cultural or sociocultural or bi-rac* or birac* or race) adj3 (status* or competen* or awareness* or diversit* or discrimin* or bias*)).ti,ab,kf AND 4. Hospices/ or “Hospice and Palliative Care Nursing”/ or Palliative Care/ or Palliative Medicine/ or exp Terminal Care/ or Terminally Ill/ or (Hospice* or Incurable or Palliative or Terminal* or End of life or Serious illness or Seriously ill or Life limit*).ti,ab,kf.
CINAHL
1. (MH “Implementation Science”) or TI ( (implement* or intervent* or qual* or program* or intermediat* or administ* or navigat*) ) OR AB ( (implement* or intervent* or qual* or program* or intermediat* or administ* or navigat*) ) AND 2. (MH “Neoplasms+”) or TI ( (cancer* or carcinoma* or carcinoid* or cyst* or lesion* or malignan* or metastas* or metastat* or neoplas* or oncol* or tumor* or tumour*) Or (adenocarcinoma* or astrocytoma* or blastoma* or carcinosarcoma* or chondrosarcoma* or choriocarcinoma* or craniopharyngioma* or ependymoma* or erythroleukem* or erythroleukaem* or fibrosarcoma* or glioblastoma* or glioma* or hepatocarcinoma* or histiocytoma* or hodgkin* or leukemi* or leukaemi* or lymphoma* or lymphosarcoma* or macroglobulinemi* or melanoma* or medulloblastoma* or meningioma* or mesothelioma* or myeloma* or myelodysplastic or myeloproliferative or nephroblastoma* or neuroblastoma* or osteosarcoma* or pheochromocytoma* or pineoblastoma* or retinoblastoma* or rhabdomyosarcoma* or sarcoma* or thymoma*) ) OR AB ( (cancer* or carcinoma* or carcinoid* or cyst* or lesion* or malignan* or metastas* or metastat* or neoplas* or oncol* or tumor* or tumour*) Or (adenocarcinoma* or astrocytoma* or blastoma* or carcinosarcoma* or chondrosarcoma* or choriocarcinoma* or craniopharyngioma* or ependymoma* or erythroleukem* or erythroleukaem* or fibrosarcoma* or glioblastoma* or glioma* or hepatocarcinoma* or histiocytoma* or hodgkin* or leukemi* or leukaemi* or lymphoma* or lymphosarcoma* or macroglobulinemi* or melanoma* or medulloblastoma* or meningioma* or mesothelioma* or myeloma* or myelodysplastic or myeloproliferative or nephroblastoma* or neuroblastoma* or osteosarcoma* or pheochromocytoma* or pineoblastoma* or retinoblastoma* or rhabdomyosarcoma* or sarcoma* or thymoma*) ) AND 3. (MH “Healthcare Disparities”) or (MH “Minority Groups”) or (MH “Health Inequities”) or TI ( (prejudice* or inequal* or inequit* or disparit* or equalit* or equit* or unequal* or injustice* or justice* or racial or racist* or ethnicit* or racism* or stigma* or sterotyp*) Or (implicit or health or conscious or unconscious or nonconscious or non-conscious) N3 (attitude* or bias*) Or (minorit* or structural* or c ultural* or multi-cultur* or multicultur* or linguistic* or social* or intercultural* or inter-cultural* or cross-cultural or crosscultural or multi-rac* or multi-ethnic* or multiethnic* or multi-lingual or multilingual or trans-cultural or transcultural or ethnocultur* or ethnocultur* or socio-cultural or sociocultural or bi-rac* or birac* or race) N3 (status* or competen* or awareness* or diversit* or discrimin* or bias*)) ) OR AB ( (prejudice* or inequal* or inequit* or disparit* or equalit* or equit* or unequal* or injustice* or justice* or racial or racist* or ethnicit* or racism* or stigma* or sterotyp*) Or (implicit or health or conscious or unconscious or nonconscious or non-conscious) N3 (attitude* or bias*) Or (minorit* or structural* or c ultural* or multi-cultur* or multicultur* or linguistic* or social* or intercultural* or inter-cultural* or cross-cultural or crosscultural or multi-rac* or multi-ethnic* or multiethnic* or multi-lingual or multilingual or trans-cultural or transcultural or ethnocultur* or ethnocultur* or socio-cultural or sociocultural or bi-rac* or birac* or race) N3 (status* or competen* or awareness* or diversit* or discrimin* or bias*)) AND 4. (MH “Hospices”) OR (MH “Palliative Care Nursing”) OR (MH “Palliative Care”) OR (MH “Hospice Care”) OR (MH “Palliative Medicine”) OR (MH “Terminal Care+”) OR (MH “Terminally Ill Patients+”) or TI ( (Hospice* or Incurable or Palliative or Terminal* or End of life or Serious illness or Seriously ill or Life limit*) ) OR AB ( (Hospice* or Incurable or Palliative or Terminal* or End of life or Serious illness or Seriously ill or Life limit*) )
Scopus
1. (implement* or intervent* or qual* or program* or intermediat* or administ* or navigat*) AND 2. (cancer* or carcinoma* or carcinoid* or cyst* or lesion* or malignan* or metastas* or metastat* or neoplas* or oncol* or tumor* or tumour*) Or(adenocarcinoma* or astrocytoma* or blastoma* or carcinosarcoma* or chondrosarcoma* or choriocarcinoma* or craniopharyngioma* or ependymoma* or erythroleukem* or erythroleukaem* or fibrosarcoma* or glioblastoma* or glioma* or hepatocarcinoma* or histiocytoma* or hodgkin* or leukemi* or leukaemi* or lymphoma* or lymphosarcoma* or macroglobulinemi* or melanoma* or medulloblastoma* or meningioma* or mesothelioma* or myeloma* or myelodysplastic or myeloproliferative or nephroblastoma* or neuroblastoma* or osteosarcoma* or pheochromocytoma* or pineoblastoma* or retinoblastoma* or rhabdomyosarcoma* or sarcoma* or thymoma*) AND 3. (prejudice* or inequal* or inequit* or disparit* or equalit* or equit* or unequal* or injustice* or justice* or racial or racist* or ethnicit* or racism* or stigma* or sterotyp*) Or ((implicit or health or conscious or unconscious or nonconscious or non-conscious) w/3 (attitude* or bias*) Or (minorit* or structural* or cultural* or multi-cultur* or multicultur* or linguistic* or social* or intercultural* or inter-cultural* or cross-cultural or crosscultural or multi-rac* or multi-ethnic* or multiethnic* or multi-lingual or multilingual or trans-cultural or transcultural or ethnocultur* or ethnocultur* or socio-cultural or sociocultural or bi-rac* or birac* or race) w/3(status* or competen* or awareness* or diversit* or discrimin* or bias*) AND 4. (Hospice* or Incurable or Palliative or Terminal* or “End of life” or “Serious illness” or “Seriously ill” or “Life limit*”)

## Appendix C. Intervention Design Categories, Category Definitions and Exemplar Quotes

**Intervention Design Category**	**Category Definitions**	**Exemplar Quotes (Study)**
**Communication Skills Programs for Patients, Caregivers, Researchers, and Clinicians**	*Programs intended to develop culturally competent communication skills for health-related end-of-life conversations among patients, caregivers, researchers, and clinicians.*	“The goal of the present study was to develop a culturally competent communication coaching intervention to promote ACP among Latino patients with advanced cancer.” (**Shen**) “We aimed to assess the acceptability of the SICG among African Americans with advanced cancer and their clinicians…The Serious Illness Conversation Guide (SICG) was developed to provide clinicians with a structured, patient-centered approach to SIC using tested language to communicate prognosis and elicit patients’ goals, values, and priorities that inform decision-making… We recruited a convenience sample of oncologists from 2 health centers to complete training in the use of the SICG.” (**Sanders**)
**Education Programs for Patients**	*Programs intended to educate patients with cancer by incorporating culturally relevant themes.*	“This study aims to adapt a video-based, multimedia chemotherapy educational intervention to meet the needs of US Latinos with advanced gastrointestinal malignancies… The intervention centers upon conversations with 12 Latino patients about their treatment experiences; video clips highlight culturally relevant themes (personalismo, familismo, faith, communication gaps, prognostic information preferences).” (**Leiter**)
**Navigation and Support Programs for Patients and Caregivers**	*Programs intended to offer personalized and culturally-tailored guidance, education, and support to patients and caregivers from specific ethnic and racial communities throughout the care journey.*	“Intervention patients received at least 5 home visits from a PN and the educational packet…Patient navigators completed detailed field notes, including tracking the number of contacts and visit length and content. The research team reviewed field notes to ensure intervention fidelity.” (**Fischer**) “In this study, we first report adapting and integrating the counseling and patient navigator interventions followed by a pilot study of the combined intervention, puente para cuidar (bridge to caring), to understand and improve the feasibility of the integrated intervention.” (**Bekelman**) “This study sought to (a) implement new volunteer support teams for African Americans with advanced cancer in two distinct regions and (b) evaluate support teams’ ability to improve support, awareness of services, and quality of life for these patients…Study participants comprised two groups—support team volunteers and persons with advanced cancer…Support team volunteers were nonprofessionals recruited from the same geographic and social communities of participating cancer patients.” (**Hanson 2014**)
**Training Programs for Health Workers and Community Leaders**	*Programs intended to equip community health workers and local leaders with the knowledge, skills, and cultural sensitivity needed to support individuals facing serious illness, promote palliative care awareness, and provide peer-based guidance within their communities.*	“Lay health advisors were recruited through African American church leaders and health ministries… Investigators delivered a day-long training, offered several times. Participants practiced approaches for communicating with, advising, and supporting persons who faced serious illness, including sensitivity to confidentiality and boundaries of peer support. Lay health advisors then met monthly for 1 year to share their outreach experiences, discuss barriers, and suggest ways to improve peer support. They also participated in discussions with hospice providers, cancer support group leaders, and supportive care professionals.” (**Hanson 2013**) “The nurse-led palliative care program, comprising 4 nurses and a medical anthropologist, trained Latino community leaders in knowledge of palliative care and advance care planning as well as how to share information about advance care planning and home-based symptom management. The palliative care lay advisors (PCLA) had to be over 18 years old, able to read and write Spanish and English, willing to assist Latinos with cancer, and have reliable transportation.” (**Johnson**) “This study introduces a novel Palliative Care Curriculum and Training Plan for CHWs [Community Health Workers], tailored to the specific needs of African American patients with advanced cancer.” (**Monton**) “Fifteen Latino community leaders completed a 10-hour palliative care training program and then served as palliative care LHAs.” (**Larson**)

## Appendix D. Intervention Tailoring for Racial and Ethnic Population Categories, Category Definitions, and Exemplar Quotes

**Intervention Tailoring Category**	**Category Definitions**	**Exemplar Quotes (Study)**
**Trust and Faith-based Values**	*Tailoring that leverages faith-based principles and values, building trust by engaging trusted community members who share the racial and ethnic backgrounds of those they serve.*	“Implementation of this peer support model was based on the socioecological theory of community health promotion using existing social networks (Golden & Earp, 2012; McLeroy, Bibeau, Steckler, & Glanz, 1988). Lay health advisors were recruited through African American church leaders and health ministries. Recruited individuals were asked to invite others whom they regarded as natural helpers. Publicity materials were included in church bulletins and in news media reaching the African American community. To be included in training, participants had to complete a short application describing personal attributes, spiritual gifts, and prior experience with serious illness and provide three references who endorsed their fitness for the lay health advisor role.” (**Hanson 2013**) “Peer support interventions are used to extend health information and practical, emotional, and spiritual support beyond health care settings (Fisher, Earp, Maman, & Zolotor, 2010). These interventions are based on the socio-ecological theory of community health promotion, which acknowledges the important role of trusted sources for health information within social networks (McLeroy, Bibeau, Steckler, & Glanz, 1988)… We developed a volunteer peer support team program for African Americans facing advanced cancer, called Circles of Care.” (**Hanson 2014**) “This study introduces a novel Palliative Care Curriculum and Training Plan for CHWs, tailored to the specific needs of African American patients with advanced cancer. The pedagogical approach and interdisciplinary nature of curriculum development can be adapted and applied to a range of healthcare domains where Community Health Workers (CHW) may be utilized to address health disparities.” (**Monton**)
**Literacy, and Faith-based Values**	*Tailoring that incorporates faith-based principles and adapts language to improve understanding and health literacy.*	“The focus group interview solicited reactions to the SICG language, questions, content, and delivery. Participants were asked about a proposed new question in the guide: “What gives you strength as you think about the future with your illness?” This question was added for piloting based on research demonstrating the importance of family support and religious faith in ACP among Black Americans and input from researchers whose focus is health disparities.” (**Sanders**)
**Language, Literacy, and Cultural Values**	*Cultural tailoring to align with patients’ language preferences, literacy levels, and core cultural values to improve understanding and engagement in care.*	“The navigators reviewed the written materials (workbook and illustrative case vignettes) of the therapy intervention and made three key recommendations: (a) revise the written materials to ensure literacy level was at the fifth- to sixth-grade reading level, (b) use videos to convey the story and core messages of the written illustrative case vignettes, and (c) adapt the vignettes to include aspects of Latina/o culture. Paired team members edited the workbook through an iterative process to achieve sixth-grade reading level, as determined by Reading IO. Next, advisory panel navigators were paired with an investigator and worked in partnership to rewrite the vignettes in the existing patient materials using surface-level and deep cultural tailoring. Surface-level cultural tailoring ensures that the people being portrayed in materials look like the people being targeted. Deep cultural tailoring includes integrating core values into the materials.”(**Bekelman**) “The objective of this study was to investigate if a culturally tailored PN [Patient Navigator] intervention can improve PC [Palliative Care] outcomes (increased ACP, improved pain management, and greater hospice use) for Latino adults with advanced cancer... All participants (control and intervention) received a culturally tailored packet of written information about ACP, pain management, hospice use, and a study-specific advance directive (AD)… An interdisciplinary, bicultural community advisory panel helped select materials…Materials were at a fifth-grade reading level and available in English and Spanish, per patient preference…Cultural Values were described in eTable 1 as utilizing core values in all messaging (cultural tailoring), Importance of family (familia or familism), Personal Connections based on trust (confianza), and value/build strong interpersonal connections (personalismo).” (**Fischer**) “The nurse-led palliative care program, comprising 4 nurses and a medical anthropologist, trained Latino community leaders in knowledge of palliative care and advance care planning as well as how to share information about advance care planning and home-based symptom management.” (**Johnson**) “The purpose of this study was to develop and evaluate a community-based palliative care lay health advisor (LHA) intervention for rural-dwelling Latino adults with cancer. Culturally relevant conversations about advance care planning began …” (**Larson**)
		“We developed a suite of videos, booklets, and websites available in English and Spanish, which convey the risks and benefits of common chemotherapy regimens. After revising the English materials, we translated them into Spanish using a multi-step process. The intervention centers upon conversations with 12 Latino patients about their treatment experiences; video clips highlight culturally relevant themes (personalismo, familismo, faith, communication gaps, prognostic information preferences) identified during the third adaptation step…Table 1 describes lowering literacy level.” (**Leiter**) “Participants were emailed a digital copy or given a hard copy (via mail or in person) of the intervention booklet in their preferred language (English or Spanish)…Major changes were made to the manual based on user feedback and included: making it easier to understand, providing supportive tables and reference materials to understand the medical jargon, simplifying the language and reducing redundancies in content, adding vignettes to give examples of the content in real-world scenarios and make it more applicable to Latinos, adding features around cultural tailoring and inclusion of family members, and providing clear end goal action steps for ACP at the end of the manual.” (**Shen**)
